# Reliability testing of a portfolio assessment tool for postgraduate family medicine training in South Africa

**DOI:** 10.4102/phcfm.v5i1.577

**Published:** 2013-11-14

**Authors:** Louis Jenkins, Bob Mash, Anselme Derese

**Affiliations:** 1 Division of Family Medicine and Primary Care, Faculty of Health Sciences, University of Stellenbosch, South Africa; 2 Western Cape Department of Health, Eden district, George Hospital, South Africa; 3 Centre for Education Development, Faculty of Medicine and Health Sciences, Ghent University, Belgium

## Abstract

**Background:**

Competency-based education and the validity and reliability of workplace-based assessment of postgraduate trainees have received increasing attention worldwide. Family medicine was recognised as a speciality in South Africa six years ago and a satisfactory portfolio of learning is a prerequisite to sit the national exit exam. A massive scaling up of the number of family physicians is needed in order to meet the health needs of the country.

**Aim:**

The aim of this study was to develop a reliable, robust and feasible portfolio assessment tool (PAT) for South Africa.

**Methods:**

Six raters each rated nine portfolios from the Stellenbosch University programme, using the PAT, to test for inter-rater reliability. This rating was repeated three months later to determine test–retest reliability. Following initial analysis and feedback the PAT was modified and the inter-rater reliability again assessed on nine new portfolios. An acceptable intra-class correlation was considered to be > 0.80.

**Results:**

The total score was found to be reliable, with a coefficient of 0.92. For test–retest reliability, the difference in mean total score was 1.7%, which was not statistically significant. Amongst the subsections, only assessment of the educational meetings and the logbook showed reliability coefficients > 0.80.

**Conclusion:**

This was the first attempt to develop a reliable, robust and feasible national portfolio assessment tool to assess postgraduate family medicine training in the South African context. The tool was reliable for the total score, but the low reliability of several sections in the PAT helped us to develop 12 recommendations regarding the use of the portfolio, the design of the PAT and the training of raters.

## Abstrait

**Tests de fiabilité d'un outil d’évaluation de portefeuille pour la formation postdoctorale en médecine familiale en Afrique du Sud**

**Contexte:**

L’éducation fondée sur la compétence ainsi que la validité et la fiabilité de l’évaluation sur le lieu de travail des étudiants en formation postdoctorale font l'objet d'une attention croissante dans le monde entier. La médecine familiale a été reconnue comme spécialité en Afrique du Sud il y a six ans et un portefeuille satisfaisant de compétences est requis pour passer l'examen national de sortie. Une augmentation massive du nombre de médecins de famille est nécessaire afin de répondre aux besoins de santé du pays.

**But:**

Le but de cette étude était de développer un outil d’évaluation de portefeuille (PAT) fiable, solide et faisable pour l'Afrique du Sud.

**Méthodes:**

Six évaluateurs ont chacun évalué neuf portefeuilles issus du programme de l'Université de Stellenbosch, en utilisant le PAT afin de tester la fiabilité inter-évaluateur. Cette évaluation a été répétée trois mois plus tard pour déterminer la fiabilité premier test/second test. À partir d'une première analyse et de commentaires, le PAT a été modifié et la fiabilité inter-évaluateur à nouveau évaluée sur neuf nouveaux portefeuilles. On a considéré qu'une corrélation > 0,80 entre les classes était acceptable.

**Résultats:**

Le score total a été jugé fiable avec un coefficient de 0,92. En ce qui concerne la fiabilité premier test/deuxième test, la différence du score total moyen était de 1,7%, ce qui n’était pas statistiquement significatif. Parmi les sous-sections, seule l’évaluation des réunions éducatives et du journal ont indiqué des coefficients de fiabilité > 0,80.

**Conclusion:**

Il s'agissait de la première tentative de développement d'un outil national d’évaluation de portefeuille fiable, solide et faisable pour évaluer la formation postdoctorale en médecine familiale dans le contexte sud-africain. L'outil était fiable concernant le score total, mais la faible fiabilité de plusieurs sections du PAT nous a aidé à développer 12 recommandations concernant l'utilisation du portefeuille, la conception du PAT et la formation des évaluateurs.

## Introduction

In 2009 the World Health Assembly resolved that it is necessary to train and retain adequate numbers of health workers with an appropriate skill mix, including family physicians (FPs), in order to respond effectively to people's health needs at the primary care level.^1^ This resolution was endorsed at the Primafamed conference in 2012 where family physicians and educators from 20 countries agreed that the family physician in Africa needs to be trained within an inter-professional primary healthcare (PHC) team and at the district hospital.^2^ Training of FPs for Africa has important differences from North America and Europe, with recent work on the principles of family medicine in Africa showing that in 70% of African settings the FP is required to perform clinical procedures and operations at the district hospital.^3,4^ At the same time, there is a growing realisation that in order to make a difference FPs must be active in PHC teams and support the development of community-orientated primary care.^5^ In South Africa, the Department of Health aims for 900 trained FPs over the next 10 years who can work within the district health system.^6^ If FPs are to provide adequate support for the PHC teams then even more FPs will be required as it is estimated that the country needs 7000 such teams. Therefore, in order to address the burden of disease in South Africa, a huge scaling up of family medicine training is being envisaged, which has relevance for appropriate postgraduate assessment.^6^

In South Africa, family medicine was recognised as a specialty in 2007 and new training programmes were aligned with a nationally-agreed set of learning outcomes.^7^ The learning outcomes were based on the competencies expected of a family physician in South Africa.^8^ Competency-based programmes in medical education are now a worldwide trend, aiming to meet such pre-defined training outcomes.^9,10,11,12,13,14^ Postgraduate students (registrars) are assessed by a unitary exit exam for postgraduate family medicine training, overseen by the Colleges of Medicine of South Africa. To improve the validity of postgraduate assessment, workplace-based assessment (WPBA) of registrars was implemented. A national learning portfolio was developed in order to capture this WPBA through a consensus process involving all eight universities in the country.^15^ This process established the content and construct validity of the portfolio. The new learning portfolio was then implemented at all universities in 2010. Registrars must show evidence of satisfactory performance, captured in this portfolio of learning over a three-year period and in an accredited training post, in order to enter the final national exit exam. Satisfactory performance is assessed and scored iteratively throughout the year by clinical supervisors utilising the WPBA tools included in the portfolio, as well as at the end of each year by the Head of Department or programme manager at each of the universities.

Portfolios of learning are being used to accumulate summative and formative assessments of performance in the workplace in many healthcare training programmes.^12,16,17,18,19,20^ In the United Kingdom, portfolios were introduced more than 20 years ago to assist in WPBA of healthcare training.^21,22^ A systematic review of the educational effects of portfolios on undergraduate medical, nursing and allied health education has shown improvements in knowledge and understanding, particularly with regard to integrating theory with practice, an increased self-awareness, engagement in reflection and improved student–mentor relationships as the main benefits of portfolio use.^22^ In other European countries, such as Belgium and the Netherlands, portfolios have been used in undergraduate and postgraduate WPBA, including high stakes judgements, for more than 10 years.^16,23^ Some universities follow an administrative process to insist on portfolio completeness, followed by an overall global rating of the contents by an assessor.^24^ Other universities have a programmatic structured scoring system of the portfolio contents by the direct supervisor, an overall supervisor and, if there is a dispute, an assessment panel.^25^

The utility of WPBA in postgraduate education, particularly its validity, reliability and feasibility, has shown mixed results. WPBA tools such as the mini Clinical Evaluation Exercise (mini-CEX) and Direct Observation of Procedural Skills (DOPS) have become standard in many postgraduate medical education programmes.^20,26,27^ A recent study looking at the composite reliability of a WPBA toolbox for postgraduate medical education in a portfolio showed that a minimum of seven mini-CEXs, eight DOPS and one multi-source feedback (MSF) was sufficient to yield reliable results.^28^ Other studies have shown mini-CEX reliability with eight to 10 assessors and DOPS reliability with two to three assessors and two cases.^20,29,30,31^ However, mini-CEX and DOPS assessments have been shown to be vulnerable to inter-rater differences.^32,33,34,35^

In view of the robustness necessary for a high stakes assessment, in this case eligibility to sit the national exit exam, we drafted a uniform portfolio assessment tool (PAT) in liaison with the eight heads of family medicine departments in the country and piloted this in the field at Stellenbosch University over a one-year period (see Addendum). The aim of this study was to establish the inter-rater reliability and test–retest reliability of the PAT. The primary objective was to establish the reliability of the total score. The secondary objective was to evaluate the reliability of the assessment of the various portfolio subsections. We also wanted to get feedback as to how to improve the assessment of the portfolios, in order to improve the feasibility and reliability of the PAT.

## Research methods and design

### Study design

This was a quantitative study that compared agreement between raters. Qualitative feedback from the raters was also collected in order to help interpret the results and make recommendations with regard to improving the feasibility and reliability. The study was conducted according to the Helsinki Declaration for research and approved by Stellenbosch University's Health Research Ethics Committee (Ref no. N09/10/258).

### Setting

The training programme at Stellenbosch University (one of the eight South African university programmes) was the setting for this study. The training programme placed registrars at one of four different training complexes. One training complex was in the metropolitan area of Cape Town and the other three were rural training complexes centred around Paarl, Worcester and George in the Western Cape. Training complexes allowed registrars to rotate between primary care facilities, district hospitals and regional and/or tertiary hospitals. In the district health services they worked under the supervision of a family physician and at regional and/or tertiary hospitals under other specialists. They worked in the complex for four years and during this time completed eight on-line modules on family medicine principles, 10 clinical family medicine domains and a research assignment.^36^ The modules focus on key family medicine principles such as the consultation, evidence-based medicine, community-orientated primary care, family-orientated primary care, ethics, leadership and governance. The clinical domains include adult health, women's health, child health, surgery, orthopaedics, anaesthetics, ear-nose-and-throat/eyes/dermatology, infectious diseases, mental health and emergency medicine.

### The learning portfolio

The postgraduate portfolio of learning starts with an introductory section that outlines the purpose of the portfolio and includes a guide to the registrars on how to build their individual portfolios. This section is followed by a section that shows which of the national outcomes are assessed in the portfolio. As the registrars meet with their supervisors, they need to ensure they have a learning plan for every clinical rotation or exposure, which is graded by the supervisor. This is followed at the end of that rotation with a supervisor report, which includes a grading score and formative feedback. An absent learning plan or supervisor report signifies an unaccounted training gap and thus an incomplete portfolio. Various types of educational meetings are counted and noted, with a minimum requirement of 24 hours per year.^37^ A minimum of 10 supervisor observations of consultations, procedures and teaching events is expected per year. These observations are graded with the help of tools such as the mini-CEX and DOPS.

Whilst every university has its own requirements for written assignments, a blueprinting exercise by the College of Family Physicians agreed on a number of topics that should always be assessed by written assignments and included in the portfolio; for example, an assignment on evidence-based medicine, community-orientated primary care and family-orientated primary care must be included. The expected skills to be captured in the logbook were agreed upon nationally and should all be covered and assessed over the four-year training period. These include 168 core skills that the registrar should be able to perform independently and 43 skills that should be performed under supervision (grey shading in the portfolio) during their training.^8^ The portfolio also has a requirement for a certificate in cardio-pulmonary resuscitation and then allows for more personal or unique entries with additional evidence of learning. At the start of every section, a summary table of the scores for that section is completed and kept updated by the registrar, to help the registrar to monitor progress and to allow for easier calculations of the aggregated scores at the end of the year. The last section shows the PAT, allowing the registrars to see how the portfolio is assessed.

### The portfolio assessment tool

We wanted to develop a feasible and reliable PAT that would allow assessment of the registrar portfolio at the end of each training year, repeated over three years, prior to application to sit the national exam in the fourth year. As most sections in the portfolio already included individual assessments with grades, the PAT was designed to aggregate these grades at the end of the year and, together with a global score by the programme manager or Head of Department, to also calculate a total score out of 100 (see Addendum). Feedback on which sections of the portfolio should be included for summative assessment was requested from registrars, supervisors and programme managers in a national survey.^38^ From this feedback a core group of experts designed the PAT to score each of these sections and added a final global assessment for the whole portfolio. The draft PAT was also discussed with the eight heads of family medicine departments in the country.

Table 1 shows how the six assessment components in the PAT correlated with the sections in the portfolio, adding up to 90 out of 100. Assessment usually involved checking whether the section had been completed and extracting any scores that had already been given. Where there were multiple scores, for example, one for each learning plan, these were added or averaged, as appropriate, to give a final score for that section. Instructions on how to do this for each section were included in the PAT. The global score, making up the last 10 out of 100 marks, assessed evidence of learning, the quality of reflections on each rotation or exposure and overall organisation of the portfolio. For the global rating, a 5-point Likert scale was developed. Instructions to the raters were given verbally during a PAT training session and supplemented with a brief written explanation in the PAT.

**TABLE 1:** A summary of the components of the portfolio assessment tool.

Sections in the portfolio	Score or grading	Description	Minimum needed
Learning plans	/10	Rating of the written learning plan by the supervisor	6-monthly; or 1 for every rotation
Rotational reports	/10	Rating of the registrar's performance by the supervisor	6-monthly; or 1 for every rotation
Educational meetings	/20	Rating of the number of hours accumulated and the range of different types of educational interactions	24 hours, 5 different types of interaction as specified in the portfolio
Observations by supervisors	/10	Rating of the registrar performing a variety of different competencies such as a consultation, procedure or teaching event	10 observations, 1 must be a teaching event
Assignments	/10	Grades obtained for written assignments	2–3 assignments per year
Logbook	/30	Rating of competency to perform clinical skills by the supervisor(A = theory only; B = have seen; C = can do under supervision;D = can do independently)	168 skills over 4 years must achieve a D rating.43 skills over 4 years must achieve at least a C rating
Global rating	/10	Rating of the overall evidence of learning, quality of reflection and organisation of the portfolio	

**Total grade**	**/100**		

### Sampling

Nine portfolios and six raters were accepted as sufficient to reveal a significant difference in grading between the raters, with 78% power to detect a 4% difference in the total score.^28^ Each rater was considered the unit of measurement, in other words, we compared raters and not portfolios. We wanted to establish the inter-rater reliability between different raters, as well as the test–retest reliability of the same raters. Six raters were selected purposively and consented to participate in the study. The six raters comprised the head of department, the postgraduate programme manager and four senior clinical trainers attached to the postgraduate programme, spread out over 500 kilometres in two training complexes. They were selected on the basis of their involvement with postgraduate training as clinical trainers or faculty members, their familiarity with the portfolio and their prior involvement with assessment of family medicine registrars. All nine first-year registrar portfolios available from 2011 were selected.

### Data collection

After a group training session for the raters, wherein a brief explanation of the PAT and a pilot study with two portfolios was given, each rater graded a copy of all nine portfolios in May 2012. The raters then graded a fresh copy of the same portfolios again in August 2012. Following initial analysis and feedback from the raters the PAT was modified and inter-rater reliability again assessed on nine new first- and second-year portfolios from 2012, in February 2013. The only modification of the PAT involved the global rating where we attempted to give clearer definitions of how reflections should be assessed for each point on the Likert scale by incorporating concepts from a published assessment model for reflections.^39^

Qualitative feedback from the raters was requested and collected by e-mail and verbal discussions. The comments were documented, collated and, if necessary, clarified with the raters. Where common issues emerged, these were considered in the subsequent modification of the PAT. These comments also helped to interpret some of the quantitative results.

## Analysis

There were three sets of analysis:

1. Inter-rater reliability testing of the PAT – comparing reliability between the six raters who each rated all nine portfolios.
2. Test–retest reliability of the PAT – comparing reliability between the initial assessments of the six raters using the PAT, as well as a follow-up assessment of the same six raters using the PAT again on the same nine portfolios.
3. Inter-rater reliability testing of the modified PAT – comparing reliability between the same six raters, each using the modified PAT to assess a different set of nine portfolios.

Inter-rater reliability was calculated using a dependant *t*-test that determined agreement between the different scores for each rater. An intra-class correlation coefficient (ICC) was calculated so as to test for consistency of assessment between raters for each section of the PAT as well as the final total score. An acceptable ICC was considered to be 0.80 or higher.

Test–retest reliability was calculated using a dependant *t*-test on the average score for each section of the PAT as well as on the average total score for each rater at baseline and three months later, given the assumptions that reliability would indicate either no differences between the test–retest scores or that the differences would be less than 4%, as explained in the sampling section. A reliable test would not be significantly different and would therefore have a *p*-value > 0.05. Spearman's correlation was also calculated between the baseline and three-month scores and a good correlation would be significant with *p* < 0.05.

## Results

### Inter-rater reliability

Table 2 illustrates the initial assessments in May 2012. A reliability coefficient > 0.80 was achieved for four of the sections in the PAT, but not for the total score. Assessing the logbook, giving a global rating and the total score had particularly low reliability coefficients. Possible reasons for this from raters’ comments included:

- Not all the registrars completed their logbooks in the same way, as some used the ‘A’ to ‘D’ system to rate their competency, whilst others used numbers; logbook entries were scattered all over the portfolios and were not always grouped together; and the grey shading that indicated the 43 skills which only required a C rating was not visible, causing confusion amongst the raters.
- The global rating elicited a very specific discussion amongst the raters. It became clear that the assessment of reflections was not easy, with difficulty in differentiating between the five categories on the Likert scale.

**TABLE 2:** Inter-rater reliability testing in May 2012.

PAT sections	Overall ICC consistency	95% CI
Learning plans (/10)	0.93	0.85–0.98
Rotation reports (/10)	0.83	0.65–0.95
Educational meetings (/20)	0.87	0.71–0.96
Observations (/10)	0.78	0.56–0.93
Assignments (/10)	0.82	0.64–0.95
Logbook (/30)	0.33	0.08–0.70
Global rating (/10)	0.51	0.24–0.82
Total score (/100)	0.58	0.31–0.85

PAT, portfolio assessment tool; ICC, intra-class correlation coefficient; CI, confidence interval.

The reliability of the total score was consequently influenced, with a low ICC of 0.58. This was particularly as a result of the low agreement on the logbook, which made up 30% of the total score.

### Test-retest reliability

Looking at the total score (/100) for the portfolio, the mean score in May was 64.2 and in August was 65.6, with a difference of 1.7%, which was not statistically significant. Table 3 illustrates that only one component, educational meetings, was rated significantly differently between May and August. The assessment of the educational meetings had two variables – firstly, the type of interaction (e.g. case discussion, setting learning agenda, indirect observation and feedback, intermittent evaluation, evidence-based practice) and secondly, the total number of hours accumulated over the year. Most registrars easily met the yearly minimum requirement of 24 hours (2 hours per month). The actual differences in the mean scores for educational meetings were small (12 out of 20 and 13 out of 20).

**TABLE 3:** Test-retest reliability results.

Modified PAT sections	Mean 1 (May)	Std Dev	Mean 2 (Aug)	Std Dev	Difference	Confidence -95%	Confidence +95%	*p*-value
Learning plans (/10)	7.66	0.36	7.75	0.35	-0.08	-0.35	0.19	0.48
Rotation reports (/10)	7.96	0.19	7.97	0.26	-0.00	-0.34	0.32	0.95
Educational meetings (/20)	12.10	0.49	13.03	0.65	-0.92	-1.57	-0.28	0.01
Observations (/10)	4.57	0.98	4.30	0.57	0.27	-0.57	1.12	0.44
Assignments (/10)	1.76	0.52	2.20	1.46	-0.43	-2.03	1.15	0.51
Logbook (/30)	23.05	1.39	23.54	1.70	-0.49	-2.71	1.73	0.59
Global rating (/10)	7.14	0.80	6.72	1.12	0.42	-0.09	0.94	0.09
Total score (/100)	64.23	2.82	65.53	3.10	-1.29	-4.30	1.70	0.31

PAT, portfolio assessment tool; Std Dev, Standard Deviation.

### Inter-rater reliability with the modified portfolio assessment tool

The two sections of concern were the calculation of the logbook score and the global rating. The grading of the different skills in the logbook (168 at grade D and 43 at grade C) was improved in the modified PAT. Table 4 illustrates the inter-rater reliability of assessments with the modified PAT in February 2013. The total score for the PAT was now found to be reliable, with a coefficient of 0.92. Overall only educational meetings, the logbook and the total score showed reliability coefficients > 0.80. Sub-analysis of the four components with low reliability coefficients indicated that one rater differed significantly from the others in the assessment of assignments, whilst more than one rater differed significantly from the others in their assessment of learning plans, rotation reports, observations and the global rating. Three factors that influenced these results were identified immediately as follows:

- For learning plans, the PAT instructions are clear, but the raters did not follow them consistently.
- For rotation reports, one registrar joined mid-year in August 2012 and therefore only required one report. Some raters did not take cognisance of this and inappropriately penalised the registrar for a missing report. The portfolio did not make this clear and thus needs to be amended to make the time from entry to the programme clearer.
- A factor causing inconsistency in calculating the mean scores for observations could have been that some portfolios had more than the minimum of 10 observations documented. Some raters graded the first 10, others the best 10, whilst yet others used all of the observations. Again, clearer PAT instructions are needed.

**TABLE 4:** The inter-rater reliability of assessments with the original and modified portfolio assessment tool.

Modified PAT sections	ICC consistency (2012)	ICC consistency (2013)	95% CI (2013)
Learning plans (/10)	0.93	0.40	0.14–0.75
Rotation reports (/10)	0.83	0.26	0.04–0.65
Educational meetings (/20)	0.87	0.89	0.75–0.97
Observations (/10)	0.78	0.21	0.00–0.60
Assignments (/10)	0.82	0.76	0.54–0.93
Logbook (/30)	0.33	0.91	0.81–0.98
Global rating (/10)	0.51	0.48	0.21–0.80
Total score (/100)	0.58	0.92	0.81–0.98

PAT, portfolio assessment tool; ICC, intra-class correlation coefficient; CI, confidence interval.

## Discussion

The final version of the PAT demonstrated a reliable total score for the assessment of the portfolio. This was largely because the components which contributed the most to the final score also demonstrated good inter-rater reliability. This pattern is similar to work from Europe on internship portfolios, where inter-rater reliability coefficients for 15 tasks ranged from 0.58 to 0.79, with a reliability coefficient of 0.89 for the instrument as a whole (95% CI = 0.83–0.93).^24^

Nevertheless the variability in the reliability coefficients forced a serious review of three areas – the rating process, the PAT itself and the way in which the portfolio was completed. Inter-rater reliability coefficients > 0.80 were achieved for four of the sections in the PAT during initial rating and for three of the sections during the subsequent rating nine months later. There was a training session with the raters prior to the initial rating in May 2012, but this was not repeated for the subsequent rating in February 2013. The drop in inter-rater consistency between the two rounds could be explained in part by this, showing the necessity of a training session prior to using the PAT, together with clearer instructions in the PAT. It is recognised that assessors often rely on assumed discriminators of performance levels, for example the difference between borderline and satisfactory and therefore need specific training in assessment processes in order to enhance reliability.^29,30,34,40^ We know that the tools are only as good as the raters using them.^41^ This is particularly true as postgraduate portfolio assessment is a very recent introduction in our programme, as well as in most of South Africa. Evidence exists that in some countries inter-rater reliability coefficients showed improvement over an eight-year period, as raters developed experience and registrars and supervisors developed clarity on expectations.^24^ We attempted to maintain maximum feasibility of the end of year assessment process in the PAT, which meant that most of the assessments have already been completed and captured during the course of the year (adding validity) and that six of the seven assessment tasks in the PAT were really more of an administrative collation or calculation based on existing scores.

The process of assessing the portfolios of registrars and testing the reliability of the PAT has given feedback on the training programme itself. At this stage we have opted for an approach of grading all the learning activities entered into the portfolio. This is a dilemma, as the risk exists that people will construct their portfolios to obtain the marks, rather than as a genuine reflection of their experience and learning. For example, in drawing up a learning plan, the goal should not be to obtain a good score, but to have a valid and practical plan for learning, as discussed between the registrar and supervisor.^42^ However, without the prompt for a score, these plans are often not drawn up or not captured and registrar–supervisor meetings may be neglected. Also, more attention is needed to make the registrars and supervisors aware of the need to match their learning plans and end-of-rotation assessments with the national training outcomes, as detailed in the introduction to the portfolio and the section containing the expected national outcomes.

Rating and doing direct observations are a recent introduction to our training programmes and many registrars and supervisors find this difficult to accomplish, as noted in several other international studies.^14,43,44,45^ The benefits are well recognised, including more valid assessment, better personal development and better patient care.^46,47^ The challenges include large workloads, lack of supervisors working close to the registrars, personal fears of taking risks and simply the change management principles of introducing something new.^42,43^ The poor scores for assignments via the PAT can be attributed to the fact that the various assignments had been scored and the grades collected by the university via another process and the registrars did not see these as being part of their portfolios. This dilemma has subsequently been corrected, including challenging the strong mindset that assignments are separate from everyday clinical work.

In rating the logbook entries many registrars scored well, even in their first year of training. This would imply either prior appropriate learning, or good training in that registrar year, or perhaps an optimistic tendency in assessment by themselves and their supervisors.^48^ The 30% contribution to the total portfolio score (Table 1) is indicative of the emphasis placed in the training of family physicians on clinical skills, which has been recognised as being essential in the African context.^3,4^ Ideally, the logbook entries should be captured in one rubric over four years, showing development and the completeness of meeting expected learning outcomes.

## Limitations and strengths

Although the study had a small sample size, we had sufficient power to detect a 4% difference in the inter-rater and test–retest reliability scores of the total grades. For improved reliability assessment of the various portfolio subsections, we would need a larger sample of portfolios, which will become available as more registrars are using them. The total mark for the portfolio is considered as one of the entry criteria for the final national examination, for which we were able to show good reliability. The wide confidence intervals in the inter-rater reliability testing results are explained in part by the small number of portfolios assessed. The low ICC scores for the different sections of the portfolio were not too surprising, considering the short track record of registrars and supervisors using the portfolio and the small number of scores per section in the analysis, but it has helped us to identify areas that are clearly in need of improvement.

### Recommendations

In discussion with the raters, a number of suggestions were made with regard to improving the reliability of the various sections, particularly in relation to:

1. Use of the portfolio:
  - Have an annual training workshop for registrars and supervisors, to ensure we enhance fidelity to the portfolio requirements and forms of assessment.
  - Ensure the registrars describe how far they are from initial registration when they submit the portfolio, as some people join mid-year.
  - Ensure the continuous rating of learning activities by the clinical supervisors and entry of these in the correct places in the portfolio to make the aggregation of scores easier at the end of the year.
  - Review the scoring of some of the tools in the portfolio (some of this has already been completed, e.g. streamlining all grades to scores out of 10).
  - Capture the iterative ratings throughout the year electronically, allowing a more streamlined administrative function and giving more continuous feedback to the registrars.
2. The design of the PAT:
  - Make the instructions clearer, for example, to calculate the grades of the 10 best observations.
  - Have uniformity with decimals and rounding, for example, the number of hours spent in educational meetings.
  - Review the global rating section, for example, to assess the quality of reflections and organisation of the portfolio in two separate Likert scales.
3. Training of raters:
  - Have an annual rater training workshop, focusing on reliable use of the PAT, particularly for new raters.
  - Develop a video clip explaining the use of the PAT that can remind raters prior to their occasional use of the tool.
4. Use of the PAT for national exam purposes:
  - Can be recommended as the total score reliability coefficient was 0.92.
  - With the above recommendations, a repeat reliability study with a larger sample of portfolios in a year will help toward improving and monitoring the reliability of the ratings of the different portfolio subsections.

## Conclusion

The aim of this study was to evaluate the reliability of a portfolio assessment tool and to improve its feasibility and reliability in assessment of the family medicine postgraduate portfolio of learning. Whilst the overall reliability coefficient of 0.92 for the total score supports its use as a tool to evaluate the portfolio, the poorer reliability of various subsections in the tool has prompted 12 recommendations for the portfolio itself, the tool and the raters.

## Appendix 1

SECTION 10 – Standard National Family Medicine Postgraduate Portfolio Assessment Tool (PAT): Annual assessment.

Three satisfactory annual portfolio scores (> 60%) are needed for verification to the CMSA that the candidate is ready for the Part 1 Exam in 4th year. The annual score will also be used by the University for its own assessment purposes. All PAT scoring can be completed by a competent administrative person as the information is already in the portfolio, assuming the HOD/Program manager has completed section 10.

1. A learning plan (section 3) for each rotation undertaken and a minimum of 2 per year. Missing learning plans should be scored as zero. If at least 2 learning plans, but one is not scored, take the average score of those scored. The learning plan is assessed in the portfolio as Excellent = 10, Satisfactory = 6, Poor = 2, Unacceptable = 0. Final score out of 10. Take the average of the scores for each learning plan as the score for the year.

**Table:**

Learning plans	First learning plan score	Second learning plan score	Third learning plan score	FINAL AVERAGE (…..../10):

2. Report/Reflection on Rotations (Section 3)**: Portfolio cannot be seen as acceptable overall if a report is missing**. In the portfolio there is a global assessment out of 10 that can be used as an overall score for the rotation. Take the average of the scores for each rotation as the score for the year.

**Table:**

Supervisor report	First report score	Second report score	Third report score	FINAL AVERAGE (…..../10):

3. Add up the number of hours recorded for educational meetings (section 4) and divide the total by 4 to give a score for the year. The max score possible is 10. In addition give 2 points for each type of meeting, if it appears at least once in the portfolio (A, B, C, D, E, F) to a max of 10. Add the two scores together to give a final score for the year out of 20.

**Table:**

Educational Meetings	Score for hours (Total hours/4) =	2 Points per category A-F	A, B, C, D, E, FScore for categories =	TOTAL (…./20):

4. Calculate the average score for the 10 required observations (section 5). Each observation should already have been scored out of 10. Missing (number less than 10) observations should be counted as zero. At least one must be a scored teaching activity.

**Table:**

Observations (each scored …../10)	1	2	3	4	5	6	7	8	9	10 (Teach)	FINAL AVERAGE (……./10)

5. Course assignments (already assessed in course out of 100%). **At least one assignment is required from each of the 5 key areas by the end of 3 years**. There should be at least one new assignment per year. An average score is calculated for all of the assignments at the end of each year. The final average score should be reduced to a score out of 10 and not 100. If an assignment marked with * is absent, score = 0 for that assignment.

**Table:**

Year 1	Ethics and medico-legal*	Evidence-based medicine*	Clinical patient study (optional)	FINAL AVERAGE (…../10)
Year 2	Quality improvement*	Community-orientated primary care*	Additional (optional)	FINAL AVERAGE (…../10)
Year 3	Family-orientated primary care*	Teaching and learning	Additional (optional)	FINAL AVERAGE (…../10)
Year 4	Elective assignment	Elective assignment	Additional (optional)	FINAL AVERAGE (…../10)

* Required by CMSA [The Colleges of Medicine of South Africa]

6. Logbook (section 7): Look at the skills in the **unshaded** blocks and add up the total number of ‘D'ratings. To give a score out of 20 divide the total number by 8 for a 4th-year registrar, 6 for a 3rd-year registrar, 4 for a 2nd-year registrar and 2 for a 1st-year registrar. Give the score to the nearest whole number and to a maximum of 20. {Please confirm on the electronic copy of the portfolio logbook which blocks are shaded or not. Photocopies are not always clear.}

Look at the skills in the **shaded** blocks and add up the total number of both ‘D’ or ‘C’ ratings. To give a score out of 10 divide the total number by 4 for a 4th-year registrar, 3 for a 3rd-year registrar, 2 for a 2nd-year registrar. Do not divide for a 1st-year registrar. Give the score to the nearest whole number and to a maximum of 10. Add the two scores together to give a final score out of 30.

**Table:**

Score for unshaded skill blocks (…./20)		Score for shaded skill blocks (…../10)		SUM OF TWO SCORES (……/30):

7.Section 10: The Program Manager will make a global rating of the portfolio (Also using the reflections on learning in section 3, and a Likert scale.)

**Table:**

	SCORE SELECTED (…......./10):

**Table:**

Reflections on rotations:^1^	
1–2 Poor	Experiences or clinical activities are described (What happened?).
3–4 Barely adequate	Essential aspects identified – thoughts, feelings and contextual factors described (Self-awareness).
5–6 Average	Critical analysis: Why and How questions and searching for answers. (Awareness of frame of ref.).
7–8 Good	Conclusions drawn – new perspectives formulated.
9–10 Excellent	Translation of new perspectives into new behaviour (concrete learning goals and plans for future actions described).
Feedback:	
1–2 Poor	Almost non-existent. Doubt about registrar's competencies. May need to repeat a rotation.
3–4 Barely adequate	Suggests a borderline registrar.
5–6 Average	Indicate no problems. Performance is OK, but no praise.
7–8 Good	Engaging registrar, exceeding expectations, contributing more than expected, standing out.
9–10 Excellent	Exceptional registrar whom the supervisor would employ.
Organisation of portfolio:	
1–2 Poor	Portfolio reads detached from real work/learning experience. Filled in mostly later in the year. Disjointed, disorganised, or incomplete.
3–4 Barely adequate	Complete, but some areas are disorganised, not showing clearly how learning happened over the course of the year.
5–6 Average	Complete and organised in a systematic way. It reads congruent with experience, filled in throughout the academic year.
7–8 Good	Complete and comprehensive and clear. It is a model example, above expectations.
9–10 Excellent	The registrar engaged the portfolio from early on in the year, consistently and systematically added items, including additional evidence like e.g. photos, videos, patient reports.

1 Koole et al. BMC Medical Education. 2011; 11:104.

**Table:**

Portfolio Assessment Tool (PAT) Score..………..../100
